# Real-world decline in survival with poor guideline adherence in chronic myeloid leukemia care

**DOI:** 10.1038/s41408-025-01423-5

**Published:** 2025-11-24

**Authors:** Sanne J. J. P. M. Metsemakers, Geneviève I. C. G. Ector, Avinash G. Dinmohamed, Rosella P. M. G. Hermens, Nicole M. A. Blijlevens

**Affiliations:** 1 Department of Hematology, Radboud University Medical Center, Nijmegen, The Netherlands; 2 Department of Internal Medicine, Rijnstate Hospital, Arnhem, The Netherlands; 3 Department of Research and Development, Netherlands Comprehensive Cancer Organization (IKNL), Utrecht, The Netherlands; 4 Department of IQ Health, Radboud University Medical Center, Nijmegen, Netherlands

**Keywords:** Haematological cancer, Health services, Prognosis

**Dear Editor,**

Chronic myeloid leukemia (CML) has shifted from a fatal to a chronic disease with tyrosine kinase inhibitors (TKIs) [1]. Its prevalence, now 10−15 per 100,000, is rising, projected to plateau by 2050 at 35 times the annual incidence, making CML one of the most common hematologic malignancies [2, 3]. Management is guided by international protocols, with adherence essential for optimizing outcomes and quality of life [4, 5]. The main goal within 12 months of TKI-therapy is achieving major molecular response, followed by deep molecular response [6]. Emerging strategies like treatment-free remission (TFR) and dose reduction require strict guideline-based management to be effective. CML care is fragmented, posing risks to optimal monitoring and guideline adherence (GA), with unfavorable impact on outcomes [3, 7, 8]. GA in CML care is often suboptimal, and its impact on survival remains unclear. Quality indicators (QIs), measurable benchmarks from clinical guidelines, are used to assess adherence and care quality in practice [9, 10]. Our study evaluates adherence to QIs and examines the impact of GA on overall survival (OS).

Data from the Netherlands Cancer Registry included all adult CML patients diagnosed 2014–2022, with first-year BCR::ABL1 monitoring data crucial for outcomes [11–13]. Data were analyzed using five QIs related to complete diagnostics (QI1), timely TKI-initiation (QI2), adequate monitoring (QI3-5), and impact on OS. Methods and quality indicators are detailed in the supplementary files and table S1.

We performed descriptive analyses of demographics and QI adherence, comparing patients with ≤2 vs. ≥3 QIs. Survival was analyzed across adherence levels using Kaplan–Meier and Cox models, adjusting for age, sex, prior malignancy, and time since diagnosis. Additional statistical analyses included Wilcoxon, Chi-Square, Shapiro-Wilk, and Schoenfeld residuals. OS was analyzed using vital status, updated annually for all patients. Progression-free survival (PFS) was not assessed due to the low number of expected progressions.

The registry included 1536 newly diagnosed chronic-phase CML patients between 2014 and 2022. Demographics are presented in supplementary Table S2. Forty-seven patients in the acute phase or blast crisis at diagnosis were excluded from the analysis. Median age was 60 years (IQR 47-70) and most patients were male (58.07%, n = 832). Mean years since diagnosis were 6.16 years (SD 2.5). 254 patients (16.54%) had a prior malignancy, while 254 (12.24%) patients developed a second malignancy after CML. After being diagnosed with chronic-phase CML, 51 patients (3.32%) subsequently progressed to accelerated or blast phase.

Table 1 shows adherence to QIs and subcategories. A total of 1214 out of 1536 patients (79.04%) adhered to QI1, reflecting diagnostic workup. QI2, regarding timely TKI-initiation, was met by 87.80% of the patients (n = 1338). QI3 was met by 590 out of 1531 (38.54%), QI4 was met by 456 out of 1521 patients (29.98%) and QI5 was met by patients 305 out of 1488 patients (20.50%).

**Table 1:** Adherence to guideline-based quality indicators (per indicator and by subcategories).

*Adherence to Quality indicator:*	*Yes (n/N)*	%
***1 (diagnosis + bone marrow)***	1214/1536	79.04
***2 (start TKI)***	1338/1524	87.80
***3 (3 months monitoring)***	590/1531	38.54
Monitoring at 3 months and good response	559/1531	36.51
If monitoring at 3 months but response failure	358/1531	23.38
Performance of a mutation-analysis within 6 weeks	43/358	12.01
In presence of mutation: TKI switch	2/2^a^	100.00
New BCR::ABL1 sampling within 2 months after failure	181/358	50.56
***4 (6 months monitoring)***	456/1521	29.98
Monitoring at 6 months and good response	419/1521	27.55
If monitoring at 6 months but response failure	342/1521	22.49
Performance of a mutation-analysis within 6 weeks	49/342	14.33
In presence of mutation: TKI switch	5/5^b^	100.00
New BCR::ABL1 sampling within 2 months after failure	166/342	48.54
***5 (1 year monitoring)***	305/1488	20.50
Monitoring at 12 months and good response	278/1488	18.68
If monitoring at 12 months but response failure	340/1488	22.85
Performance of a mutation-analysis within 6 weeks	38/340	11.18
In presence of mutation: TKI switch	5/5^c^	100.00
New BCR::ABL1 sampling within 2 months after failure	141/340	41.47

Adherence to the monitoring quality indicators was met if monitoring was conducted within three months and patients showed a good response. Alternatively, if there was no response within three months, adherence was maintained if a mutation analysis was performed. If the mutation analysis is positive, a TKI switch and follow-up BCR::ABL1 monitoring should be performed within two months of the initial sampling to ensure adherence to quality indicators.

*TKI* Tyrosine Kinase Inhibitor

^a^2 patients had a positive mutation analysis, 38 had a negative result, and 3 had unknown results.

^b^5 patients had a positive mutation analysis, 41 had a negative result, and 3 had unknown results.

^c^5 patients had a positive mutation analysis, 26 had a negative result, and 7 had unknown results.

Figure 1A provides an overview of the number of QIs adhered to by patients. A small percentage of patients, 2.51% (n = 37), did not adhere to any of the QIs. Full adherence to all five QIs was observed in 5.62% (n = 83). Figure S1 shows highest QI adherence in patients aged <20 and lowest in those aged >80. However, these age groups are small (n = 5 and n = 120) compared to other groups (n = 229, 526, 656).

**Fig. 1:**
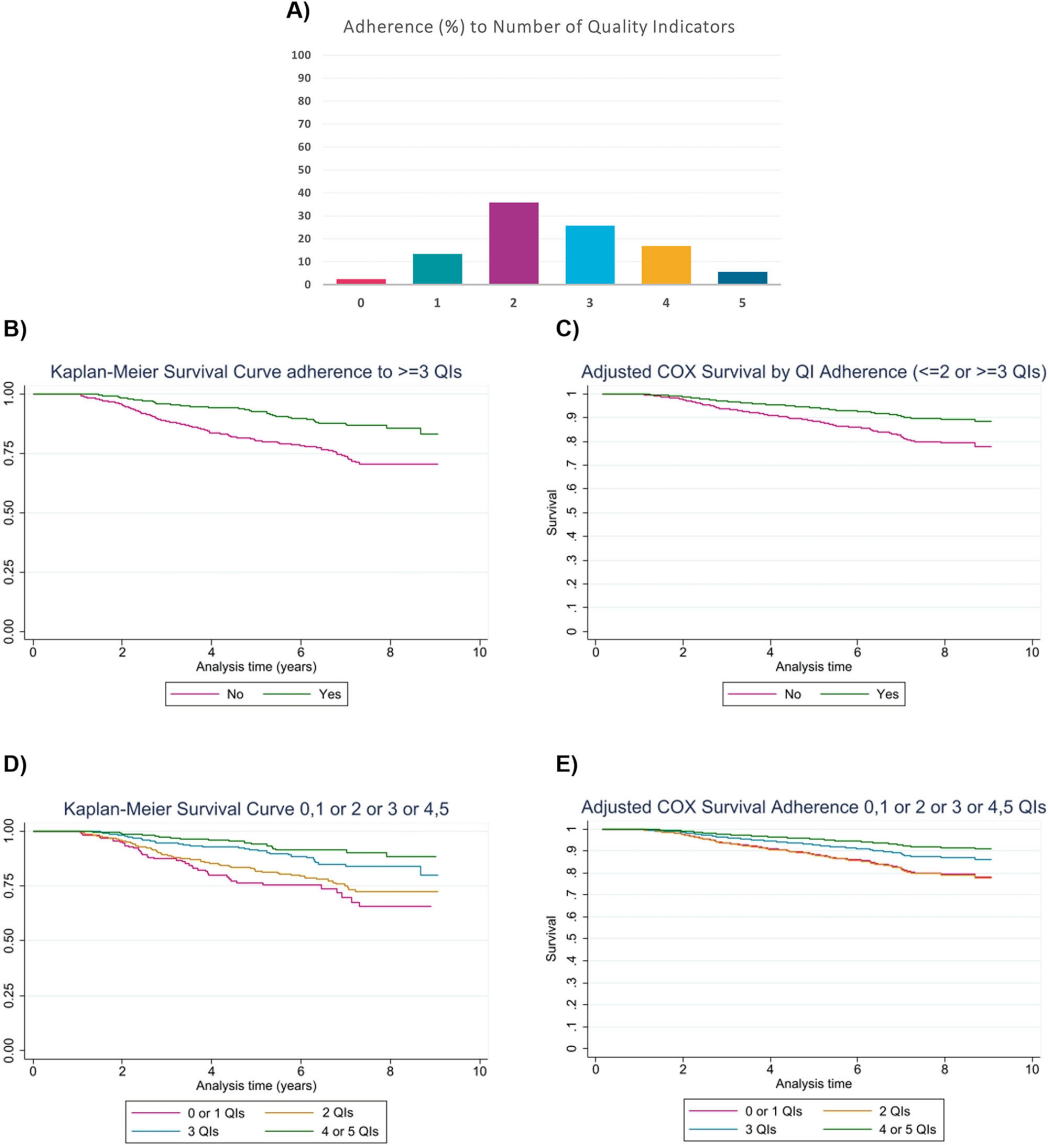
Combined visualization of study outcomes: Adherence to number of quality indicators and Kaplan–Meier survival analyses. **A** Percentage of patients adhering to zero, one, two, three, four or five Quality Indicators (n = 1477). **B** Kaplan–Meier overall survival curve comparing patients adhering to two or less or three or more QIs. **C** COX proportional hazard model curve comparing patients adhering to two or less or three or more QIs, adjusted for age, sex, years since diagnosis and prior malignancy. **D** Kaplan−Meier overall survival curve comparing four groups of patients: those adhering to zero or one QIs, two QIs, three QIs, and four or five QIs. **E** COX proportional hazard model curve comparing four groups of patients: those adhering to zero or one QIs, two QIs, three QIs, and four or five QIs, adjusted for age, sex, years since diagnosis and prior malignancy.

We compared baseline characteristics of patients adhering to ≤2QIs with those adhering to ≥3QIs. Patients adhering to ≥3QIs were significantly younger (median: 57, IQR 45–68) than patients adhering to ≤2QIs (median: 62, IQR 50–72). The mean years since diagnosis varied between the groups: 6.32 years (SD 2.51) for patients adhering to ≥3QIs, and 6.03 years (SD 2.50) for patients adhering to ≤2QIs. Prior malignancy was observed less frequently in patients adhering to ≥3QIs (n = 98, 13.74%) than in those with adherence to ≤2QIs (n = 139, 18.19%). There was a slight, non-significant difference in progression to advanced disease between patients adhering to ≥3QIs (2.66%) and those adhering to ≤2QIs (3.40%). WHO performance status was largely missing. Among available data, WHO 0 was most common, followed by 1 and 2. Differences between ≤2 and ≥3QI groups were statistically significant but not substantial, and interpretation is limited by missing data. No significant differences were found in sex, ELTS (EUTOS long-term survival) score, or later malignancies between the groups.

Figure 1B shows the Kaplan–Meier curve for patients adhering to ≤2QIs and patients adhering to ≥3QIs, showing OS is better for patients adhering to ≥3QIs. For these groups, the Cox model yields a hazard ratio (HR) of 0.41 (95% CI: 0.30–0.56), indicating a 59% lower risk of death for patients adhering to ≥3QIs compared to those adhering to ≤2QIs. After adjusting for age, sex, years since diagnosis, and prior malignancy (Fig. 1C), the HR remains favorable at 0.50 (95% CI: 0.36–0.68).

Figure 1D presents the Kaplan–Meier curve for patients adhering to zero or one, two, three, or four or five QIs, illustrating improved OS with better adherence. Relative to 0-1 QIs, unadjusted HRs were 0.76 (95% CI: 0.53–1.11) for 2QIs, 0.42 (0.27–0.65) for 3QIs, and 0.26 (0.15–0.44) for 4-5QIs. After adjusting for age, sex, time since diagnosis, and prior malignancy, HRs were 1.02 (0.70–1.50), 0.61 (0.39–0.96), and 0.38 (0.22–0.66), respectively. Figure 1E presents the adjusted Cox curves.

While sex and years since diagnosis do not significantly impact the HR, older age and prior malignancy are associated with higher risk of death.

In this Dutch real-world study, adherence to guideline-based QIs was low. Highest adherence was for TKI-initiation (87.80%) and diagnosis (79.04%), while monitoring adherence was lower (38.54%, 29.98%, 20.50% at 3, 6, and 12 months). Only 83 patients (5.62%) adhered to all five QIs; most adhered to two (35.82%) or three (25.73%). Results show that GA to more QIs is associated with better OS. Patients who adhered to ≥3QIs had a 59% lower risk of death compared to patients ≤2QIs. After adjusting for age, sex, years since diagnosis, and history of prior malignancy, the risk remained 50% lower. Patients were grouped in more detail by QI adherence: adjusted mortality was similar for 0–2 QIs, 39% lower for three QIs, and 62% lower for four or five QIs, emphasizing the importance of GA for outcomes. No significant difference in progression to advanced disease was observed between patients adhering to ≥3 versus ≤2QIs, likely reflecting the small number progressions. Higher BCR::ABL1 levels, however, may prompt stricter follow-up and improved guideline adherence. PFS was not assessed due to the limited number of progressions.

Findings of the current study align with the study of Ector et al. which also showed low adherence to QIs in Dutch practice [10]. Similarly to the current study, only six percent of patients in that study met all QIs. Milojkovic et al. evaluated treatment and monitoring patterns in the UK, revealing frequent gaps in molecular assessments [14]. Although studies on overall CML GA are scarce, some specifically examine monitoring. Goldberg et al. reported that 3–4 annual monitoring tests reduced progression risk and improved PFS [12]. Under-monitoring of treatment response may hinder detection of resistance or suboptimal response, limiting timely TKI adjustments and compromising outcomes, as described by Saleh et al. [15].

Adequate GA allows early detection of TKI intolerance or resistance, enabling timely treatment adjustments and better outcomes. Despite clear recommendations, adherence remains low, suggesting feasibility issues or gaps in awareness among patients and hematologists. Identifying barriers and understanding these challenges is essential for improving adherence. Centralizing care in specialized centers could improve GA and outcomes, supported by collaboration and patient education to promote self-management.

Strengths of this study include the use of real-world data, inclusion of all patient groups (including elderly), a large cohort of over 1500 CML patients, and assessment of survival impact. These features enhance generalizability, robustness, and demonstrate the link between GA and patient outcomes, offering valuable insights beyond typical clinical trials. Study limitations include missing patient characteristics, e.g., ELTS scores, which restricted adjustment of survival analyses. Data on comorbidities, causes of death, and treatment-related toxicities were unavailable, limiting comprehensive OS analysis. The study focused on GA during the first year post-diagnosis, the critical period for achieving major molecular response [13, 16, 17]. Monitoring milestones were assessed with a ± 14-day margin, a practical choice supported by prior research, as no specific window is described in the guidelines [10]. We assessed margins of 2, 7, and 21 days, which affected adherence percentages. Smaller margins led to fewer patients meeting criteria. Smaller margins changed adherence rates but did not affect overall findings, survival remained higher in patients adhering to ≥3QIs.

We applied the guidelines in place at the time. New 2024 Dutch guidelines are expected to cause minimal changes, since core aspects remain consistent. Emerging goals like TFR and dose reduction may reduce TKI use, side effects, and costs, and improved GA could increase the number of patients eligible for these strategies.

In conclusion, adherence to guideline-based QIs in Dutch real-world CML care is low, particularly for monitoring, and most patients meet only two or three QIs. Better GA is associated with improved OS, highlighting the crucial role of GA in optimizing patient outcomes and supporting emerging treatment goals like TFR and dose reduction.

## Supplementary information

Supplementary Files - REAL-WORLD DECLINE IN SURVIVAL WITH POOR GUIDELINE ADHERENCE IN CHRONIC MYELOID LEUKEMIA CARE

## Data Availability

All data is openly available and can be requested through the Netherlands Cancer Registry of the Netherlands Comprehensive Cancer Organization.
