# Supplementary material for: Real-world decline in survival with poor guideline adherence in chronic myeloid leukemia care
Source: Blood Cancer J. 2025 Nov 24;15(1):205. doi: 10.1038/s41408-025-01423-5 (PMC12641029; doi:10.1038/s41408-025-01423-5)
Supplement: Supplementary file 1 — Supplementary Files - REAL-WORLD DECLINE IN SURVIVAL WITH POOR GUIDELINE ADHERENCE IN CHRONIC MYELOID LEUKEMIA CARE [file 41408_2025_1423_MOESM1_ESM.docx]

## Supplementary files - Blood Cancer Journal

## Title

## Real-World Decline in Survival with Poor Guideline Adherence in Chronic Myeloid Leukemia CARE

## Authors and Affiliations

Sanne J.J.P.M. Metsemakers MSc, Department of Hematology, Radboud University Medical Center, Nijmegen, Netherlands

Dr. Geneviève I.C.G. Ector, Department of Internal Medicine, Rijnstate Hospital, Arnhem, Netherlands

Dr. Avinash G. Dinmohamed, Department of Research and Development, Netherlands Comprehensive Cancer Organization (IKNL), Utrecht, Netherlands

Prof. dr. Rosella P.M.G. Hermens, Department of IQ Health, Radboud University Medical Center, Nijmegen, Netherlands*

Prof. dr. Nicole M.A. Blijlevens, Department of Hematology, Radboud University Medical Center, Nijmegen, Netherlands*

*These authors contributed equally to this work and share last authorship.

## Corresponding Author

Sanne Metsemakers, MSc

Department of Hematology, Radboud University Medical Center

Geert Grooteplein Zuid 8, Nijmegen, 6525 GA, Netherlands

Email: Sanne.Metsemakers@radboudumc.nl

## Supplementary methods

**Data, Setting & study population**

This study was performed within a Dutch healthcare setting. Data was gathered from the Netherlands Cancer Registry (NCR), a real-world population-based registry maintained by the Netherlands Comprehensive Cancer Organization (IKNL), which collects data on newly diagnosed cancer patients in all Dutch hospitals. IKNL collects this data as commissioned by the Ministry of Health, Welfare and Sport.

The authors requested to use this data, and approval for its use was granted by the ethical board of IKNL. The dataset included data on all newly diagnosed CML patients aged ≥18 years in the Netherlands, diagnosed between January 2014 and January 2022. Monitoring data, i.e. BCR::ABL1 molecular testing results, from the first year after diagnosis were available, aligning with the fact that the first year’s monitoring endpoints are most critical for patient outcomes^1,2^. Survival status and dates are up-to-date, as these are annually updated in the registry. IKNL ensures updated survival statuses and dates by linking the registry to Statistics Netherlands (CBS) for continuous survival data updates. Causes of death are not included in the data. The registry further contains data on demographics, diagnosis, TKI initiation, laboratory outcomes, mutation analyses, prior malignancies, and WHO performance status.

In the Netherlands, all residents are covered by mandatory health insurance, ensuring universal access to healthcare services. There are no restrictions on access to tyrosine kinase inhibitors (TKIs), as these treatments are fully reimbursed under the national health insurance system.

**Quality indicators**

QIs of CML care were adopted from the existing research of Ector et al^3^, in which QIs were generated based on the recommendations of the European LeukemiaNet 2020 and the Dutch HOVON Guidelines ^4^. These guidelines are comparable to the American National Comprehensive Cancer Network (NCCN) guidelines; however, response milestones differ^5,6^. Since this study is performed in the Netherlands, guideline adherence and the QIs was based on European and Dutch guidelines. The QIs are shown in table 1. One QI was based on diagnosis, one on initiation of TKI treatment, and three indicators based on the strict monitoring recommendations of the guidelines. We followed the guidelines that were in effect at the specific time patients entered the care pathway. The core components related to diagnosis, TKI treatment, and monitoring remained consistent across all guideline versions.

QI 1 consisted of the percentage of patients completing diagnostic testing. The essential criteria for diagnosis include leukocytosis in peripheral blood and the detection of the Philadelphia chromosome, t(9;22)(q34;q11), and/or the BCR::ABL fusion gene through cytogenetic analysis and/or molecular testing. Additionally, a bone marrow exam is recommended^4,7^. The second indicator referred to the percentage of patients receiving a first-line TKI (imatinib, dasatinib, nilotinib or bosutinib) within 28 days after diagnosis. This timeframe is based on the 'Treek’ norms, which are standardized criteria established by the Dutch government for clinical practice and data collection in healthcare. These criteria help ensure consistency and alignment with national standards for treatment and patient management^8-10^. QIs three, four, and five are monitoring (cytogenetic or BCR::ABL1) indicators, reflecting guideline-defined milestones at specific timepoints^4,7,11^. In case of milestone failure within such a monitoring indicator, adherence was further evaluated by performance of mutation analyses, TKI switch if a mutation has been found and a new molecular BCR::ABL1 test two months after initial milestone failure. The timeframe for mutation analysis was six weeks following a failed response, with an additional ten days for the results to become available (totaling 52 days). For conducting a new molecular test, the timeframe was two months plus seven days. If any of these steps were not followed, the QI was not adhered to. Monitoring QIs three, four, and five are in line with the expected monitoring performance at three, six and twelve months since start of TKI treatment. These indicators allowed a maximum deviation of 14 days, either earlier or later, from the intended three, six, or twelve months. Patients were included in the analysis of an indicator only if they were alive at the time it was (supposed to be) performed.

*Table S1 Description of the Quality Indicators.*

|  | **Diagnosis** | |
| --- | --- | --- |
| **Indicator 1** | % Of patients with complete diagnostic and molecular cytogenic workup at diagnosis | |
|  | **Treatment** | |
| **Indicator 2** | % Of patients receiving first-line TKI within 28 days after diagnosis | |
|  | **Follow-up** | |
| **Indicator 3-5** | % Of patients receiving BCR::ABL1 (IS) or cytogenetic^a^ monitoring at defined milestones and in case of milestone failure: | |
|  | - Performance of a mutation-analysis within six weeks^b^ - In presence of mutation: TKI switch - New BCR::ABL1 sampling within two months after failure^c^ | |
|  | **Indicator 3** | At 3 months +/- 14 days |
|  | **Indicator 4** | At 6 months +/- 14 days |
|  | **Indicator 5** | At 12 months +/- 14 days |

*^a^ Cytogenetic monitoring only an option at 3 or 6 months as defined in guidelines.*

^b^ *With a margin of an additional 10 days.*

^c^ *With a margin of an additional 7 days.*

**Data analysis**

Descriptive analyses were performed to evaluate demographics and adherence to each QI. Wilcoxon rank-sum Test and Chi-Square Test were used to evaluate baseline differences between groups. Shapiro–Wilk was used to test for normality of data. Patients adhering to two or less QIs were compared to those adhering to three or more. Additionally, survival outcomes were analyzed across adherence levels (0–5 QIs). If categories were too small, they were combined as needed. Overall survival was assessed using the Kaplan-Meier method with log-rank test. A *p* value lower than 0.05 was considered significant. Cox Proportional Hazard Models were used to evaluate OS hazard ratios corrected for potential confounding variables, such as age, years since diagnosis, presence of prior malignancy and sex. Schoenfeld residual test was used to evaluate the proportional hazards assumption. Analyses were performed using STATA/SE 17.0 (StataCorp LLC).

## Supplementary results

*Table S2 Demographics of the total population (n=1536) compared to patients adhering to ≤2 QIs (n=764) and ≥3 QIs (n=713).*

|  |  | Total (n=1536) | Adhering to ≤2 QIs (n=764) | Adhering to ≥3 QIs (n=713) | P-values |
| --- | --- | --- | --- | --- | --- |
| Age, Median (IQR) | | 60 (47-70) | 62 (50-72) | 57 (45-68) | **0.000**^a^ |
| Sex, n (%) | |  |  |  |  |
|  | **Male** | 892 (58.07) | 450 (58.90) | 397 (55.68) | 0.211^b^ |
|  | **Female** | 644 (41.93) | 314 (41.10) | 316 (44.32) |  |
| Incidence year,  Median (Min-Max) | | 2018 (2014-2022) | 2018 (2014-2022) | 2018 (2014-2022) | **0.027^a^** |
| Years since diagnosis,  Mean (Std Dev) | | 6.16 (2.50) | 6.03 (2.50) | 6.32 (2.51) | **0.027^a^** |
| First-Line TKI, n (%) | |  |  |  |  |
|  | **Imatinib** | 1202 (78.26) | 620 (81.15) | 536 (75.18) | **0.007**^b^ |
|  | **Dasatinib** | 202 (13.15) | 96 (12.57) | 98 (13.74) |  |
|  | **Nilotinib** | 125 (8.14) | 47 (6.15) | 76 (10.66) |  |
|  | **Bosutinib** | 4 (0.26) | 1 (0.13) | 3 (0.42) |  |
|  | **Unknown/Missing** | 3 (0.2) | 0 (0) | 0 (0) |  |
| ELTS Score, n (%) | |  |  |  |  |
|  | **Low-Risk** | 56 (3.65) | 28 (3.66) | 28 (3.93) | 0.780^b^ |
|  | **Intermediate-Risk** | 84 (5.47) | 46 (6.02) | 36 (5.05) |  |
|  | **High-Risk** | 48 (3.13) | 23 (3.01) | 20 (2.81) |  |
|  | **Missing** | 1348 (87.76) | 667 (87.30) | 629 (88.22) |  |
| Performance Status, n (%) | |  |  |  |  |
|  | **WHO 0** | 508 (33.01) | 234 (30.63) | 236 (36.89) | **0.019**^b^ |
|  | **WHO 1** | 215 (14.00) | 105 (13.74) | 105 (14.73) |  |
|  | **WHO 2** | 32 (2.08) | 16 (2.09) | 8 (1.12) |  |
|  | **WHO 3** | 3 (0.20) | 3 (0.39) | 0 (0) |  |
|  | **WHO 4** | 2 (0.13) | 2 (0.26) | 0 (0) |  |
|  | **Unknown/Missing** | 776 (50.52) | 404 (52.88) | 337 (47.27) |  |
| Prior Other Malignancy, n (%) | |  |  |  |  |
|  | **Yes** | 254 (16.54) | 139 (18.19) | 98 (13.74) | **0.020**^b^ |
|  | **No** | 1282 (83.46) | 625 (81.81) | 615 (86.26) |  |
| Later Other Malignancy, n (%) | |  |  |  |  |
|  | **Yes** | 188 (12.24) | 92 (12.04) | 90 (12.62) | 0.734^b^ |
|  | **No** | 1348 (87.76) | 672 (87.96) | 623 (87.38) |  |
| Disease progression after diagnosis, n (%) | |  |  |  |  |
|  | **Yes** | 51 (3.32) | 26 (3.40) | 19 (2.66) | 0.402^b^ |
|  | **No** | 1482 (96.48) | 735 (96.20) | 694 (97.34) |  |
|  | **Missing** | 3 (0.20) | 3 (0.39) | 0 (0) |  |

*^a^ Wilcoxon rank-sum (Mann–Whitney) test*

*^b^ Chi-squared test*

Schoenfeld residual test did not show violation of the proportional hazard assumptions. Varying the QI groupings did not affect the results. Similarly, adjusting for first-line TKI as a potential confounder had no impact on the findings.

Table S3 Adherence by Number of Monitoring Quality Indicators (QI3–5).

| Compliance to number of monitoring QIs | Frequency | % |
| --- | --- | --- |
| 0 (Compliance t no monitoring QIs) | 662 | 44.49 |
| 1 | 425 | 28.56 |
| 2 | 284 | 19.09 |
| 3 (Compliance to all monitoring QIs) | 117 | 7.86 |
| Total | 1488 | 100.00 |

*Figure S1. Percentage of patients adhering to QI1 to QI5 by age category (total n = 1477). The distribution of patients by age category was as follows: 18–20 years, n = 5; 20–40 years, n = 229; 40–60 years, n = 526; 60–80 years, n = 656; and ≥80 years, n = 120.*

## supplementary files references

1 Goldberg, S. L. *et al.* Association between molecular monitoring and long-term outcomes in chronic myelogenous leukemia patients treated with first line imatinib. *Current medical research and opinion* **29**, 1075-1082 (2013).

2 Goldberg, S. L. *et al.* First‐line treatment selection and early monitoring patterns in chronic phase‐chronic myeloid leukemia in routine clinical practice: SIMPLICITY. *American journal of hematology* **92**, 1214-1223 (2017).

3 Ector, G. I. *et al.* Adherence to quality indicators in chronic myeloid leukemia care: results from a population-based study in The Netherlands. *Leukemia & Lymphoma* **64**, 424-432 (2023).

4 Hochhaus, A. *et al.* European LeukemiaNet 2020 recommendations for treating chronic myeloid leukemia. *Leukemia* **34**, 966-984 (2020).

5 Narlı Özdemir, Z., Kılıçaslan, N. A., Yılmaz, M. & Eşkazan, A. E. Guidelines for the treatment of chronic myeloid leukemia from the NCCN and ELN: differences and similarities. *International journal of hematology* **117**, 3-15 (2023).

6 Shah, N. P. *et al.* Chronic myeloid leukemia, version 2.2024, nccn clinical practice guidelines in oncology. *Journal of the National Comprehensive Cancer Network* **22**, 43-69 (2024).

7 Leukemie, R. C. M. *et al.* RICHTLIJN CHRONISCHE MYELOIDE LEUKEMIE© 2018 Stichting Hemato-Oncologie voor Volwassenen Nederland (HOVON) HOVON centraal bureau VU medisch centrum. (2018).

8 Gijsberts, A., Berkers, P., van Dijk, D. & Moeke, D. Wachten in de zorg. (2019).

9 Hutten, J. B., Beaujean, D. J. & Groenewegen, P. P. De zorg van medisch specialisten. *Utrecht (Nederland): NIVEL* (2003).

10 Zagt, A., Friele, R., De Jong, J. & Bos, N. Zorggebruikers weten dat er schaarste ontstaat in de gezondheidszorg. *Utrecht: Nivel* (2023).

11 Janssen, J. *et al.* Richtlijnen voor de behandeling van chronische myeloïde leukemie anno 2014. *Ned Tijdschr Hematol* **11**, - (2014).
